# Feasibility study of temporary permanent pacemaker in patients with conduction block after TAVR

**DOI:** 10.3389/fcvm.2023.978394

**Published:** 2023-01-25

**Authors:** Sanshuai Chang, Xinmin Liu, Zhi-Nan Lu, Jing Yao, Chengqian Yin, Wenhui Wu, Fei Yuan, Taiyang Luo, Ran Liu, Yunfeng Yan, Qian Zhang, Junzhou Pu, Thomas Modine, Nicolo Piazza, Hasan Jilaihawi, Zhengming Jiang, Guangyuan Song

**Affiliations:** ^1^Interventional Center of Valvular Heart Disease, Beijing Anzhen Hospital, National Clinical Research Centre for Cardiovascular Diseases, Capital Medical University, Beijing, China; ^2^UMCV, Hôpital Haut Leveque, Centre Hospitalier Universitaire (CHU) de Bordeaux, Bordeaux, France; ^3^Montreal and German Heart Centre, McGill University Health Center, Munich, Germany; ^4^NYU Langone Health, New York, NY, United States; ^5^Department of Cardiology, The First Affiliated Hospital of Zhengzhou University, Zhengzhou, Henan, China

**Keywords:** TAVR, atrioventricular block (AV block), permanent pacemaker (PPM), LBBB, left bundle branch block

## Abstract

**Background:**

Limited data exist on the use of temporary permanent pacemaker (TPPM) to reduce unnecessary PPM in patients with high-degree atrioventricular block (HAVB) after transcatheter aortic valve replacement (TAVR).

**Objectives:**

This study aims to determine the feasibility of TPPM in patients with HAVB after TAVR to provide prolonged pacing as a bridge.

**Materials and methods:**

One hundred and eleven consecutive patients undergoing TAVR were screened from August 2021 to June 2022. Patients with HAVB eligible for PPM were included. TPPM were used in these patients instead of conventional temporary pacing or early PPM. Patients were followed up for 1 month. Holter and pacemaker interrogation were used to determine whether to implant PPM.

**Results:**

Twenty one patients met the inclusion criteria for TPPM, of which 14 patients were third-degree AVB, 1 patient was second-degree AVB, 6 patients were first degree AVB with PR interval > 240 ms and LBBB with QRS duration > 150 ms. TPPM were placed on the 21 patients for 35 ± 7 days. Among 15 patients with HAVB, 26.7% of them (*n* = 4) recovered to sinus rhythm; 46.7% (*n* = 7) recovered to sinus rhythm with bundle branch block. The remains of 26.7% patients (*n* = 4) still had third-degree AVB and received PPM. For patients with first-degree AVB and LBBB, PR interval shortened to < 200 ms in all 6 patients and LBBB recovered in 2 patients. TPPM were successfully removed from all patients and no procedure-related adverse events occurred.

**Conclusion:**

TPPM is reliable and safe in the small sample of patients with conduction block after TAVR to provide certain buffer time to distinguish whether a PPM is necessary. Future studies with larger sample are needed for further validation of the current results.

## Introduction

Transcatheter aortic valve replacement (TAVR) has rapidly evolved over the last 20 years as a definitive therapeutic option for patients with severe aortic valve diseases. Improvements in valve design, valve deployment technologies, preprocedural imaging and increased operating experience have led to a decline in procedural complications. While the incidence of post-TAVR high-degree atrioventricular block (HAVB) and new-onset left bundle-branch block (LBBB) has decreased to some extent, conduction disturbance remains the major complication (1).

During the procedure of TAVR, conduction disturbances result primarily from a direct mechanical insult to the conduction system associated with various degrees of edema, hematoma, and ischemia, as demonstrated by necropsy studies (2). Various studies have revealed most consistent predictors of permanent pacemaker (PPM) implantation after TAVR included pre-existing right bundle branch block (RBBB), pre-existing first degree AVB, lower implantation depth, high calcium load below the coronary cusp, self-expanding valve, and prosthesis oversizing (3–5). However, previous studies have demonstrated < 50% long-term pacemaker dependency rates after TAVR (6–8), which suggested that TAVR induced HAVB may be reversible and resolve over time (1), as a result, it is not always apparent immediately after the procedure which patients with new-onset conduction abnormalities ultimately require PPM. Further monitoring of these patients is helpful.

In 2013, European guidelines recommend that a 7-day monitor to be used as a threshold for whether to install PPM (9), while in the 2021 European guidelines, permanent pacemaker can be used in patients with HAVB that persists for 24–48 h after TAVR (10), the new recommendation was based on an observational study of only 50 patients (11). Prospective trials to investigate surveillance and management of HAVB after TAVR are lacking and current guidelines are based mostly on expert opinions (12).

A prolonged observation period implies the use of a temporary pacemaker with bedrest. Prolonged temporary pacing brings dangers for bleeding, infection, thromboembolism, and cardiac perforation. Those complications caused by over 24 h temporary pacing might dilute potential benefits from temporary pacing. Thus, the safety, efficacy, and cost-effectiveness and the impact on functional recovery among post-TAVR AVB patients should be considered.

To circumvent the conundrum, temporary permanent pacemaker (TPPM) involving an active fixation pacing lead connected to an external pulse generator was used to provide a longer and safer bridging period in patients with infected cardiac implantable electronic devices undergoing lead extraction (13). However, the safety and efficacy of this approach have not been thoroughly investigated in patients with conduction block after TAVR. In this study, we investigated the efficacy and safety of TPPM in patients with conduction block after TAVR.

## Materials and methods

### Study population

This study was conducted in Beijing Anzhen hospital and the First Affiliated Hospital of Zhengzhou University. All consecutive patients who underwent TAVR from August 2021 to June 2022 were screened. Patients who developed conduction block with indications for PPM received a TPPM for 4 weeks as a bridge. The study was reviewed and approved by the institutional review board at Beijing Anzhen Hospital and patients provided informed written consent for participation.

High-degree AVB (HAVB) was defined as any of the following: second degree atrioventricular block (AVB) type 2 (Mobitz II) in the presence of a QRS ≥ 120 ms; 2:1 AVB in the presence of a QRS ≥ 120 ms; ≥ 2 consecutive P waves at a constant physiologic rate that do not conduct to the ventricles; complete heart block (CHB) defined as P waves with a constant rate with dissociated ventricular rhythm (no association between P waves and R waves) or fixed slow ventricular rhythm in the presence of atrial fibrillation.

### Data acquisition, follow up, and endpoints

All patients were followed for 4 weeks after TPPM. Baseline demographics, comorbidities, type and size of the valve, Electrocardiograms (ECGs) before and after TAVR, during TPPM procedure, every week after TPPM were traced for each patient. Pacemaker interrogation, including ventricular pacing rate (VPR), was obtained from clinical visits at each follow-up, pacemaker was initially set as VVI model with a rate of 60 beats per minute (bpm), for patients with recovery of HAVB and VPR < 10%, the lower rate limit of pacemaker declined by 10 bpm every week, to reduce unnecessary pacing during nocturnal sinus bradycardia.

The primary endpoint of the present analysis was the success rate of TPPM removal and free from PPM at 4 weeks after TPPM. The criteria of removing TPPM was no indication of permanent pacing and no pacing signal in 12 lead ECG and 24-h ambulatory ECG, meanwhile the last pacemaker interrogation indicated 0% VPR.

### TPPM procedure

**FIGURE 1:**
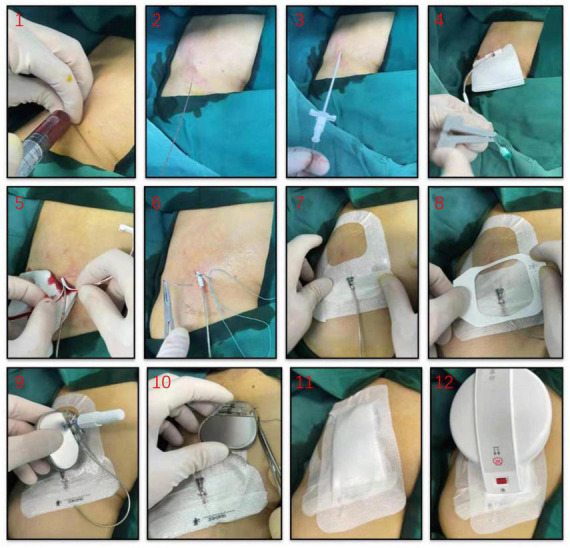
Illustrative diagram for temporary permanent pacemaker (TPPM) procedure. An active-fixation, single-chamber pacemaker lead is fixated to the right ventricular septum, the lead’s suture sleeve was sutured to the skin, and a pulse generator was connected to the lead and placed over the patient’s skin using an adhesive dressing.

Patients were temporarily paced using a permanent active-fixation lead (St. Jude Medical, 2088TC, US) permitting bipolar stimulation. The lead was inserted through a 7 or 8 F peel-away introducer sheath, the electrode positioned in the right ventricular septum with the proximal end fixed to the skin surface using the suture sleeve and connected to a pulse generator (e.g., Medtronic, St. Jude Medical) to allow prolonged temporary VVI pacing. The pacemaker was also secured by sutures in on skin surface next to the lead implantation site using an adhesive dressing (Figure 1). The pacing threshold was accepted when acute measurements demonstrated stable ventricular capture at < 1 V/0.48 ms and sensing values > 5 mV. To prevent loss of capture, the output was set to 3.5 times the documented pacing threshold. The sensitivity value was set at half the sensing threshold.

### Statistical analysis

Statistical analyses were performed using IBM SPSS Statistics for Windows version 25.0 (IBM, Armonk, New York, USA). Continuous variables were expressed as mean ± standard deviation. Categorical data are represented as frequencies and percentages, and differences between groups were evaluated using the chi-square test. Logistic regressions were used to estimate the independent effect of multiple variables on 30-day conduction recovery. The results of such analysis are presented as odds ratios (ORs) and 95% CIs. *P*-value < 0.05 was considered statistically significant.

## Results

**FIGURE 2:**
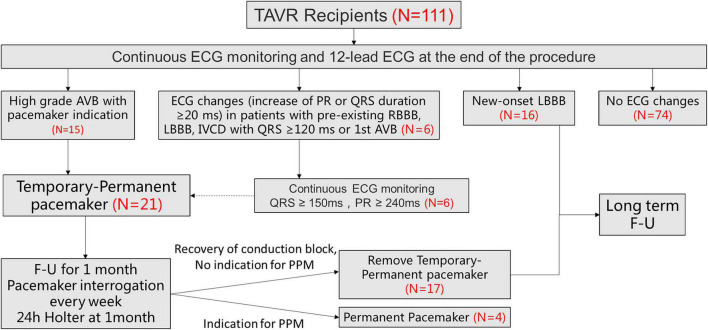
Patient flowchart. The overall population included 111 patients who underwent TAVR screened for TPPM. 21 patients ultimately underwent TPPM including 15 with HAVB and 6 with further ECG changes (increase of PR or QRS duration ≥20 ms, meanwhile QRS > 150 ms and PR >240 ms). TAVR, transcatheter aortic valve replacement; ECG, electrocardiogram; HAVB, high-degree atrioventricular block; LBBB, left bundle-branch block; TPPM, temporary permanent pacemaker; F-U, follow up.

Among 111 consecutive patients undergoing TAVR between August 2021 and June 2022, 21 patients met the inclusion criteria (Figure 2). Of those, 15 patients were third degree atrioventricular block (AVB), 1 patients was second degree AVB with obvious dizziness, 5 were first degree AVB (PR interval > 240 ms) with left bundle branch conduction block (LBBB) (QRS duration > 240 ms).

Baseline demographics of the study population were presented in Table 1. TAVR procedure and TPPM procedure characteristics were summarized in Table 2. TPPM were placed at patients immediately after TAVR (57.1%) and were placed at remain patients within 7 days after TAVR.

**TABLE 1:** Baseline characteristics according to the use of temporary permanent pacemaker (TPPM).

	Total (*N* = 111)	Without TPPM (*N* = 90)	With TPPM (*N* = 21)	*P*-value
Age (y)	72.3 ± 8.6	71.6 ± 8.3	75.4 ± 9.4	0.062
Female	48.6%	50.0%	42.9%	0.555
RBBB	6.3%	5.6%	9.5%	0.861
I°AVB	9.9%	6.7%	19.0%	0.173
AR	18.0%	14.4%	33.3%	0.043
Hypertension	68.5%	67.8%	71.4%	0.746
Diabetes	28.8%	33.3%	9.5%	0.030
CAD	38.7%	40.0%	33.3%	0.572
CVD	13.5%	15.6%	4.8%	0.193
CKD	12.6%	12.2%	14.3%	0.726
COPD	5.4%	3.3%	14.3%	0.080
PCI	22.5%	23.3%	19.0	0.672
Cardiac surgery	6.3%	7.8%	0%	0.343

Values are mean, n (%). 1° AVB, first-degree atrioventricular block; RBBB, right bundle branch block; AS, aortic valve stenosis; AR, aortic valve regurgitation; CAD, coronary artery disease; CKD, chronic kidney disease; COPD, chronic obstructive pulmonary disease; PCI, percutaneous coronary intervention; other abbreviations as in Figure 2.

**TABLE 2:** Transcatheter aortic valve replacement (TAVR) and temporary permanent pacemaker (TPPM) procedure characteristics of each patient.

	Valve disease			TAVR procedure		TPPM procedure				
	AS	AR	Annulus	Balloon	Valve type/Annulus	TPPM timing	Vascular access	Threshold	Operation	X ray
1	Severe	Mild	20.9 mm	20 mm	Self-expanding/23.5 mm	During TAVR	Right jugular	0.5 v	20 min	9 mGy
2	Severe	Moderate	21.2 mm	18 mm	Self-expanding/25 mm	6 days after TAVR	Left axillary	0.5 v	25 min	14 mGy
3	Moderate	Moderate	21.1 mm	20 mm	Self-expanding/23 mm	During TAVR	Right jugular	0.75 v	18 min	8 mGy
4	Severe	Moderate	22.4 mm	20 mm	Self-expanding/23.5 mm	During TAVR	Right axillary	0.75 v	40 min	23 mGy
5	Severe	Mild	23 mm	22 mm	Self-expanding/26 mm	During TAVR	Right subclavian	0.5 v	15 min	9 mGy
6	–	Severe	22.8 mm	–	Self-expanding/26 mm	2 days after TAVR	Right axillary	0.75 v	32 min	12 mGy
7	Moderate	Severe	27.5 mm	–	Self-expanding/32 mm	4 days after TAVR	Right axillary	0.5 v	25 min	11 mGy
8	Severe	Severe	23.7 mm	22 mm	Self-expanding/25 mm	4 days after TAVR	Right axillary	0.75 v	21 min	15 mGy
9	Severe	Mild	21.6 mm	20 mm	Self-expanding/23.5 mm	During TAVR	Right axillary	0.5 v	26 min	10 mGy
10	Severe	Moderate	27 mm	22 mm	Self-expanding/26 mm	During TAVR	Right subclavian	0.75 v	22 min	13 mGy
11	Severe	Moderate	24.1 mm	20 mm	Self-expanding/25 mm	3 days after TAVR	Right subclavian	0.75 v	25 min	17 mGy
12	Severe	Severe	21.1 mm	18 mm	Self-expanding/23.5 mm	During TAVR	Right axillary	0.75 v	21 min	25 mGy
13	Severe	Mild	26 mm	22 mm	Self-expanding/26 mm	During TAVR	Right axillary	0.5 v	32 min	28 mGy
14	Severe	Moderate	26.7 mm	21 mm	Balloon-expanding/27 mm	4 days after TAVR	Right axillary	0.75 v	35 min	17 mGy
15	Moderate	Severe	25 mm	–	Self-expanding/32 mm	During TAVR	Right axillary	0.25 v	35 min	19 mGy
16	Severe	Mild	22 mm	18 mm	Self-expanding/23.5 mm	During TAVR	Right jugular	0.6 v	20 min	8 mGy
17	Severe	Mild	20.9 mm	18 mm	Self-expanding/23.5 mm	During TAVR	Right subclavian	0.7 v	29 min	21 mGy
18	–	Severe	28.5 mm	–	Self-expanding/32 mm	4 days after TAVR	Right axillary	0.25 v	35 min	19 mGy
19	Moderate	Severe	25 mm	–	Self-expanding/32 mm	6 days after TAVR	Right axillary	0.5 v	38 min	22 mGy
20	–	Severe	25.8 mm	–	Self-expanding/32 mm	2 days after TAVR	Left axillary	0.5 v	20 min	13 mGy
21	–	Severe	24.9 mm	–	Self-expanding/32 mm	5 days after TAVR	Right subclavian	0.8 v	24 min	15 mGy

Abbreviations as in Figure 2.

**FIGURE 3:**
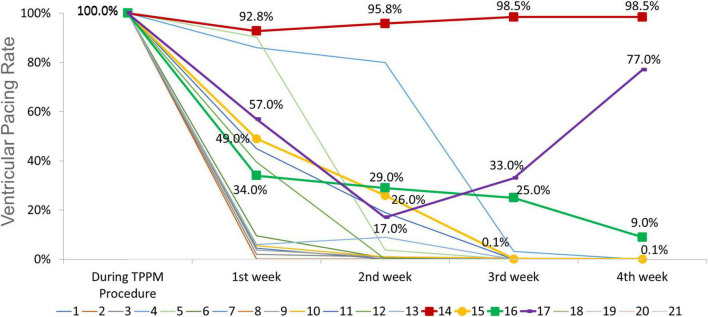
Trends of ventricular pacing rate during follow up for each individual (central illustration). The ventricular pacing rate (VPR) in patient no. 14 and no. 17 remained high throughout the follow-up period, the electrocardiogram (ECGs) and Holter demonstrated persistent third-degree atrioventricular block (AVB). The VPR decreased from 100 to 0.1% in patient no. 15 and from 100 to 9% in patient no. 16, however, Holter indicated intermittent third-degree AVB. These four patients received permanent pacemaker (PPM). For other 11 patients, the VPR decreased to 0%, and the ECGs and Holter revealed no indication for PPM. For patients with 1st degree AVB and left bundle branch block (LBBB), the VPR remained 0% throughout follow up.

Temporary permanent pacemakers (TPPMs) were in place for 35 ± 7 days. The improvement of conduction disturbances was presented in Table 3 and trends of ventricular pacing rate were shown in Figure 3. Among 15 patients with HAVB, 26.7% (*n* = 4) patients recovered to from conduction block; 46.7% (*n* = 7) patients were converted to bundle branch block. There was no indication for PPM according to ECG, Holter and pacemaker interrogation among the above two group patients, thus TPPM was successfully removed. Only 4 patients with HAVB after TAVR eventually received PPM after 4 weeks of TPPM, 2 of them had persistent third-degree AVB throughout the 4 weeks follow-up; the other two patients had intermittent third-degree AVB in Holter, both patients received PPM. In patients with 1st degree AVB and LBBB, PR interval shortened to < 200 ms in all 6 patients and LBBB recovered in 2 patients, thus PPM was unnecessary.

**TABLE 3:** Changing process of conduction disturbances during follow up for each individual.

	TPPM procedure			1st week			2nd week			3rd week			4th week		
	Rhythm	PR (ms)	QRS (ms)	Rhythm	PR (ms)	QRS (ms)	Rhythm	PR (ms)	QRS (ms)	Rhythm	PR (ms)	QRS (ms)	Rhythm	PR (ms)	QRS (ms)
1	3rd AVB	–	148	1st AVB + LBBB	200	133	SR + LBBB	182	142	SR + LBBB	163	141	SR + LBBB	160	144
2	3rd AVB	–	160	1st AVB	201	110	SR	165	105	SR	165	105	SR	160	102
3	3rd AVB	–	189	SR + RBBB	173	151	SR + RBBB	166	125	SR	160	115	SR	156	105
4	3rd AVB	–	172	3rd AVB	–	157	SR + LBBB	182	160	SR + LBBB	178	169	SR + LBBB	185	158
5	3rd AVB	–	164	3rd AVB	–	152	1st AVB + LBBB	220	130	SR + LBBB	182	140	SR	192	110
6	2nd AVB	300	161	SR + RBBB	184	130	SR + RBBB	173	133	SR + RBBB	165	125	SR + RBBB	161	121
7	1st AVB + LBBB	256	184	1st AVB + LBBB	340	178	1st AVB + LBBB	202	171	SR + LBBB	187	175	SR + LBBB	179	171
8	1st AVB + LBBB	265	186	1st AVB + LBBB	266	188	1st AVB + LBBB	279	186	1st AVB + LBBB	210	150	SR + LBBB	192	120
9	3rd AVB	–	158	SR + RBBB	129	132	SR + RBBB	128	127	SR + RBBB	125	132	SR + RBBB	134	127
10	3rd AVB	–	165	SR + LBBB	182	145	SR + LBBB	186	140	SR + LBBB	176	160	SR + LBBB	175	162
11	3rd AVB + AF	–	157	1st AVB + LBBB	226	176	1st AVB	251	110	AF	–	109	1st AVB	209	108
12	3rd AVB	–	155	SR + LBBB	221	150	SR + LBBB	177	144	SR + LBBB	168	144	SR + LBBB	153	141
13	3rd AVB	–	191	1st AVB + RBBB	251	172	1st AVB + RBBB	230	172	AF + RBBB	–	159	AF + RBBB	–	157
14	3rd AVB	–	205	3rd AVB	–	173	3rd AVB	–	173	3rd AVB	–	170	3rd AVB	–	178
15	3rd AVB	–	198	3rd AVB	–	202	SR + RBBB	188	152	1st AVB	253	111	3rd AVB	–	152
16	3rd AVB	–	165	1st AVB + LBBB	295	133	3rd AVB	–	108	3rd AVB	–	106	3rd AVB	–	108
17	3rd AVB	–	155	3rd AVB	–	159	3rd AVB	–	169	3rd AVB	–	159	3rd AVB	–	162
18	1st AVB + LBBB	296	158	1st AVB	260	152	1st AVB	220	112	1st AVB	205	102	SR	196	98
19	1st AVB + LBBB	250	170	1st AVB + LBBB	160	131	SR + LBBB	170	157	SR + LBBB	167	149	SR + LBBB	165	159
20	1st AVB + LBBB	288	168	1st AVB + LBBB	271	173	1st AVB	230	131	1st AVB	205	89	SR	191	91
21	1st AVB + LBBB	260	165	1st AVB + LBBB	240	158	1st AVB + LBBB	242	155	1st AVB + LBBB	221	165	SR + LBBB	150	125

SR, sinus rhythm; AVB, atrial ventricular block; RBBB, right bundle branch block; other abbreviations as in Figure 2.

As for the recovery time of conduction block, 7 of 21 patients recovered within 24 h post-TAVR, 10 of the remaining 14 patients recovered between 24 h and 1-month post-TAVR, 4 patients were implanted with permanent pacemakers after 1-month follow-up (Table 4).

**TABLE 4:** Recovery of conduction block and indication of pacemaker (PM) during different timeframes.

	End of TAVR procedure	From end of procedure to 24 h post-TAVR	From 24 h to 1-month post-TAVR
Conduction block recovery	0	7	10
Rate of conduction block recovery	0/21	33.3% (7/21)	71.4% (10/14)*
PM indication	21	14	4
Rate of PM indication	18.9% (21/111)	12.6% (14/111)	3.6% (4/111)

PM, pacemaker; TAVR, transcatheter aortic valve replacement. *The denominator is the number of patients who did not recover at 24 h post-TAVR.

With the univariate analysis of age, baseline ECG, aortic root calcifications, Implantation depth under NCC (non-coronary cusp), ΔMSID (difference between implantation depth and membranous septum length) and oversizing rate of implanted valve, no above factors could statistical significantly predict conduction recovery for the given small sample size (Table 5).

**TABLE 5:** Univariate analysis of predictors of conduction recovery after transcatheter aortic valve replacement (TAVR).

	Conduction recovery (*n* = 17)	PM dependency (*n* = 4)	Univariate		
			OR	CI	*P*-value
Age (y)	74.9 ± 9.4	77.8 ± 10.2	1.035	(0.918, 1.168)	0.575
Baseline RBBB (%)	2 (11.8%)	0 (0%)	–	–	–
Baseline I°AVB (%)	2 (11.8%)	2 (50.0%)	7.500	(0.645, 87.193)	0.107
Severe LCC calcification (%)	6 (35.3%)	2 (50.0%)	1.833	(0.204, 16.512)	0.589
Severe NCC calcification (%)	7 (41.2%)	3 (75.0%)	4.286	(0.366, 50.197)	0.246
Severe RCC calcification (%)	6 (35.3%)	2 (50.0%)	1.833	(0.204, 16.512)	0.589
Calcifications under LCC (mm)	2 (11.8%)	0 (0%)	–	–	–
Implantation depth under NCC (mm)	5.3 ± 2.5	6.8 ± 1.5	1.289	(0.816, 2.037)	0.276
ΔMSID (mm)	2.5 ± 1.9	3.5 ± 1.4	1.462	(0.689, 3.100)	0.322
Oversizing of aortic annulus (%)	13.3 ± 8.4	12.1 ± 11.6	0.187	(0, 90585.542)	0.802

ΔMSID indicates difference between implantation depth and membranous septum length; LCC, left coronary cusp; NCC, non-coronary cusp; RCC, right coronary cusp; OR, odds ratio; CI, confidence interval; other abbreviations as before.

There were no procedure-related complications including infection, lead dislodgements, or perforation noted in the 21 patients who received TPPM.

Further follow up (129 ± 55 days) with ECG revealed no PPM indication after TPPM removal in all 17 patients.

## Discussion

This prospective study confirms that TPPM using a bipolar active-fixation lead connected to a pulse generator fixed at body surface was feasible in patients with conduction block after TAVR. The results showed that TPPM were reliable in achieving temporary pacing while awaiting for possible recovery of post-TAVR conduction abnormalities and reducing unnecessary PPM for those patients who could recover from HAVB. This study also showed the TPPM was safe during the 4-weeks pacing and there were no complications occurred during the entire follow up.

High-degree AVB (HAVB) may be mitigated by PPM within 30 days after TAVR, and no adverse effect was detected in the incidence of 30-day heart failure readmissions or all-cause mortality. However, PPM may have a deleterious effect on left ventricular ejection fraction and increase the risk of heart failure or all-cause readmissions at the midterm to long-term follow-up in the latest systematic review (1, 14). At a median follow-up of 4 years, PPM was also associated with a higher risk of heart failure rehospitalization and the combined endpoint of death or heart failure rehospitalization (15). Thus, the decision to place a PPM after TAVR must be carefully considered.

Prior studies have demonstrated that a significant portion of patients who had PPM after TAVR had low pacemaker utilization during the follow-up period. The results of pacing requirement have varied among studies with inconsistent pacemaker indications, pacemaker dependency algorithm and different follow-up period (6–8). Costa et al., showed that 145 patients undergoing PPM within 30 days after TAVR, the dependency rates for pacing were 35.7, 35.8, and 33.3% at 1, 6, and 12 months, respectively (7). In the REPRISE III Trial, pacemaker dependency was dynamic (30 days: 43%; 1 year: 50%) and not consistent for individual patients over time (8). The longest follow-up study including 322 patients receiving PPM within 30 days post-TAVR, neve, up to 13.7% patients exhibited pacing < 1% of the time during a median follow-up of 4 years (15).

These results indicated the direct injury during TAVR procedure inflicted upon the conduction tissue may sometimes play a temporary role in the occurrence of the conduction abnormality. As seen in post-mortem examinations, the function of the conduction bundle branch may be impaired by peri-procedural edema and inflammation (2). These pathologic phenomena are by nature transient and may explain both the occurrence of conduction abnormalities and its spontaneous recovery. Other factors are episodes of hypotension and ischemia during TAVR (16).

Interestingly, the time to implant PPM was found to be associated with PM-dependent (7). However, the exact periods for waiting PPM are not consistent at different centers. Nevertheless, some patients probably underwent too short a period of observation before PPM and would be benefit from a longer period of watchful waiting to avoid inappropriate implants.

At present, temporary pacing is mainly used in perioperative period of TAVR. Patients must remain on telemetry and bed rest until lead removal to avoid the risk of leads displacement. Temporary cardiac pacing with passive fixation leads has also been related to a higher incidence of infection and venous thrombosis, delayed recovery, and increased length of hospital stay (17).

Therefore, for patients with conduction abnormalities after TAVR, a more reliable pacing technology is needed to achieve stable pacing protection, improve postoperative patient activity, and reduce in-hospital stay and unnecessary PPM. From this point of view, the use of TPPM plays its superiority. The advantages of the active fixation lead are the retractable helix and the extraordinary flexibility, as well as an extended scope of regular monitoring and interrogation of pulse generators in comparison with temporary external pacemakers. Vascular access of internal jugular vein or subclavian vein also permits rapid ambulation postoperative and no increase in length of hospital stay, which ensures greater patient comfort and mobility.

Over the last couple of decades, TPPM have become increasingly used in patients with infected cardiac implantable electronic devices after transvenous lead extraction. A review including 24 studies with 770 patients reported 2.3% TPPM-related infections, and loss of capture was documented in only 1%, the duration of TPPM usage varied from a few days up to 336 days (18). The consensus document of European Heart Rhythm Association also recommended TPPM with ipsilateral active fixation strategy in patients requiring antibiotic treatment before re-implantation (19).

Moreover, studies have compared the cost-effectiveness of TPPM. Although the initial cost of active fixation lead is higher, the added reliability and safety of the TPPM allowed early discharge instead of cardiac care unit monitoring. This resulted in cost equivalence at 18 h and potentially cost saving beyond 24 h (20).

Recently, TPPM has also been used in patients with conduction block after TAVR, Goncalves et al. conducted a retrospective analysis of 114 patients (21), TPPM were routinely placed prior to arterial access and valve deployment in all patients, it was left in place on average for 4.4 days, permanent pacemaker was implanted in ten patients (9%) with conduction abnormality persisted for at least 24 h. Given the retrospective nature of the study and no follow up data, it is not possible to analyze whether there were patients who had a HAVB after TAVR with spontaneous recovery. So, the focus was feasibility and safety. No access site complications, lead dislodgments or infections occurred, all patients were able to ambulate after the procedure without delay. Beyond that, our prospective study went further by extending follow up period to allow more time for resolution of conduction abnormalities prior to PPM. Moreover, with 4 weeks bridge of TPPM, a rather low rate of PPM (2%) was achieved without adverse events in our center.

Another retrospective study included 1,130 patients underwent TAVR (22). Eighty-two (7.3%) patients went directly to PPM due to complete AV block, whereas 69 patients (6.1%) had TPPM with conduction abnormalities that do not meet conventional criteria for PPM placement. Indications for TPPM included transient complete heart block < 30 min, 2nd degree AVB, Mobitz I, new LBBB with QRS > 120 ms, pre-existing RBBB, 1st degree AVB. TPPM were placed for 2.3 ± 2.4 days. Among those patients, 44.8% received PPM during the index hospitalization. The results suggested in low-intermediate risk patients, TPPM also safely provides a time period for further assessment and may prevent unnecessary PPM implantation.

To our knowledge, the present study is the first prospective study to evaluate the safety and feasibility of TPPM in post-TAVR patients with complete AVB and indication for PPM. TPPM offers an excellent chance for prolonged and stable temporary pacing to minimize PPM.

In our practice, patients receiving TPPM can be discharged early with normal daily activities and were instructed to avoid collision or friction at the TPPM location. These patients would wear transcutaneous patches with heart rate monitoring, which could be transmitted through mobile phone and checked by doctors remotely. These Patients were also asked to return to the outpatient clinic every week for 12-lead ECG, pacemaker interrogation and wound dressing changing. Active electrode positioned in the right ventricular septum was fixed to the skin surface using the suture sleeve to avoids dislodgement, while standard aseptic disinfection procedures and regular wound dressing changing were effective to minimize the risk of infection (Figure 1). Through these measures no infection, lead dislodgement, or perforation noted in this study.

### Study limitations

First, the number of patients in this study is limited and patients were recruited from only two centers. The small cohort is insufficient to definitively assess the safety of TPPM, however, the standardized procedures could be replicated and extended for larger population. The further studies are requested for further validation of the current results. Second, there was no head-to-head comparison to other methods of temporary back-up pacing after TAVR. Third, patients included in this study mainly received self-expanding valves and the differences between valve types in the recovery of HAVB could not be analyzed. Last, TPPM were placed for about 1 month in all patients, long term follow-up for these patients is not available, patients who recovered from HAVB or developed late-onset HAVB later than 1 month could not be recognized and analyzed. However, conduction abnormalities that occur more than 30 days after TAVR are rare phenotypes, and it becomes more difficult to directly link a TAVR procedure as a cause of PPM as time passes.

## Conclusion

Temporary permanent pacemaker (TPPM) with bipolar active-fixation leads and external pulse generators provide an important option for prolonged temporary pacing as a bridge to recovery or PPM for patients with conduction block after TAVR. Further studies with larger population analyzing predictors of conduction recovery after TAVR are warranted, and recommendation for timing of PPM implantation after TAVR should be proposed on a safe and necessary basis.

## Data availability statement

The raw data supporting the conclusions of this article will be made available by the authors, without undue reservation.

## Ethics statement

The studies involving human participants were reviewed and approved by the Institutional Review Board at Beijing Anzhen Hospital. The patients/participants provided their written informed consent to participate in this study. Written informed consent was obtained from the individual(s) for the publication of any potentially identifiable images or data included in this article.

## Author contributions

SC: trial design, trial conduct, and drafting of the manuscript. XL: trial design and interpretation of data. Z-NL: trial conduct and revising of the manuscript. JY, CY, WW, FY, TL, RL, YY, QZ, and JP: trial conduct and data collection. TM, NP, and HJ: interpretation of data and revising of the manuscript. ZJ: trial design, trial conduct, and coordination. GS: trial design, trial conduct, interpretation of data, and revising of the manuscript. All authors contributed to the article and approved the submitted version.
